# Cognitive interference processing in adults with childhood craniopharyngioma using functional magnetic resonance imaging

**DOI:** 10.1007/s12020-021-02824-9

**Published:** 2021-07-22

**Authors:** Daniel Svärd, Cecilia Follin, Sigridur Fjalldal, Robin Hellerstedt, Peter Mannfolk, Johan Mårtensson, Pia Sundgren, Eva Marie Erfurth

**Affiliations:** 1 Department of Diagnostic Radiology, Lund University, Lund, Sweden; 2 Department of Medical Imaging and Physiology, Skåne University Hospital, Lund, Sweden; 3 Department of Oncology, Skåne University Hospital, Lund, Sweden; 4 Department of Endocrinology, Skåne University Hospital, Lund, Sweden; 5 Department of Psychology, University of Cambridge, Cambridge, UK; 6 Department of Logopedics, Phoniatrics and Audiology, Lund University, Lund, Sweden; 7 Lund University BioImaging Center, Lund University, Lund, Sweden

**Keywords:** Craniopharyngioma, Cognitive interference, fMRI, Multi-source interference task

## Abstract

**Purpose:**

To assess cognitive interference processing in adults with childhood craniopharyngioma (CP), with and without hypothalamic injury, respectively, in terms of behavioral performance and functional magnetic resonance imaging (fMRI) activity, using the multi-source interference task (MSIT).

**Methods:**

Twenty-eight CP patients (median age 34.5 [29.0–39.5] years) were investigated at median 20.5 (16.3–28.8) years after treatment with surgical resection and in some cases additional radiotherapy (*n* = 10) and compared to 29 matched controls (median age 37.0 [32.5–42.0] years). The subjects performed the MSIT during fMRI acquisition and behavioral performance in terms of response times (ms) and accuracy performance (%) were recorded.

**Results:**

The MSIT activated the cingulo-fronto-parietal (CFP) attention network in both CP patients and controls. No differences were found in behavioral performance nor fMRI activity between CP patients (interference effect 333.9 [287.3–367.1] ms and 3.1 [1.6–5.6]%, respectively) and controls (309.1 [276.4–361.0] ms and 2.6 [1.6–4.9]%). No differences were found in behavioral performance nor fMRI activity between the two subgroups with (332.0 [283.6–353.4] ms and 4.2 [2.3–5.7]%, respectively) and without hypothalamic injury (355.7 [293.7–388.7] ms and 2.1 [1.0–5.2]%, respectively), respectively, and controls.

**Conclusion:**

Adults with childhood CP performed cognitive interference processing equally well as controls and demonstrated no compensatory fMRI activity in the CFP attention network compared to controls. This was also true for the two subgroups with and without hypothalamic injury. The results can be useful to better characterize this condition, and to optimize treatment and support for these individuals.

## Introduction

Craniopharyngioma (CP) is a rare, benign, embryonic pituitary tumor with an aggressive growth pattern and high recurrence rate, associated with increased mortality and morbidity in cardiovascular disease [1–6]. Adults with childhood CP, especially patients with hypothalamic involvement, are also at risk of cognitive impairment with deficits in memory, attention, and processing speed, even on complete hormone replacement therapy [7]. These deficits are thought to be due to both the tumor itself and its treatment, including surgical removal and additional cranial radiotherapy (CRT), leading to hypothalamic injury [8]. It has been hypothesized that the focal hypothalamic lesion induces changes in hypothalamic networks through the processes of diaschisis, i.e., altered function of an neuroanatomical structure due to damage in another remotely connected structure, and/or transneuronal degeneration, i.e., neuronal degeneration due to damage of nearby neurons, which may also contribute to the cognitive impairment in these individuals [9].

A previous study using magnetic resonance imaging (MRI) voxel-based morphometry have demonstrated reduced gray and white matter volumes in the limbic areas connected to the hypothalamus and an association between impaired long-term memory and reduced gray matter volumes in the posterior cingulate cortex in adolescents with childhood CP [10]. Another previous study using diffusion tensor imaging (DTI), reported an association between microstructural white matter alterations in the dorsal cingulum and a decline in episodic visual memory, visuospatial abilities, executive function, attention, and processing speed in adults with childhood CP [11]. Further, this study also reported an association between alterations in the ventral cingulum and a decline in episodic visual memory, and an association between alterations in the uncinate fasciculus and a decline in semantic memory [11].

Blood-oxygen level dependent functional MRI (fMRI) is based on the magnetic susceptibility of blood. Alterations in the MRI signal arise due to local changes in blood oxygenation, flow, and volume from the metabolism associated with neuronal activity. Neuroimaging studies on CP patients are scarce and, to our knowledge, only two previous studies have used fMRI to investigate functional brain alterations in relation to cognitive function in childhood CP patients [12, 13]. However, both studies had relatively small sample sizes and the follow-up time after treatment was relatively short.

The multi-source interference task (MSIT) can be used to test cognitive interference processing, which is the ability to be attentive to goal-relevant information and at the same to be able to reject goal-irrelevant information. This has been shown to reliably activate the cingulo-fronto-parietal (CFP) attention network, which includes the dorsal anterior cingulate cortex, the dorsal anterior midcingulate cortex, and the dorsolateral prefrontal cortex that is involved in target detection, novelty detection, error detection, decision-making, response selection, and stimulus/response competition [14, 15]. These regions are all partially connected through the white matter tracts previously investigated in CP patients [11].

Our aim was to use the MSIT during fMRI to assess cognitive interference processing in terms of behavioral performance and fMRI activity in adults with childhood CP. In addition, we wanted to investigate whether hypothalamic injury had any impact on this. We hypothesized that CP patients would exhibit longer response times, lower accuracy performance, and altered fMRI activity in the CFP attention network compared to controls. Furthermore, we suspected that these differences would be more pronounced in patients with hypothalamic injury.

## Materials and methods

### Study population

Sixty-four CP patients from the Southern Region of Sweden (population 2.5 million), who were treated at Lund University Hospital between 1958 and 2010, were invited to participate in the study. Excluded subjects (*n* = 23) were either assessed to be too ill (meningioma *n* = 1, neuromuscular disease *n* = 1, living in a home for disabled *n* = 2), too busy (*n* = 6), investigations too stressful according to patients (*n* = 2), had aneurysm clip (*n* = 1), did not give any reason (*n* = 7), had missing medical records (*n* = 1) or did not reply (*n* = 2). Five subjects had to withdraw from MRI examination due to presence of either a shunt causing significant artifacts (*n* = 1), pacemaker (*n* = 1), claustrophobia (*n* = 2), or weight restrictions (*n* = 1). A total of 36 subjects completed MRI examination. One subject was excluded due to difficulties in understanding how to perform the MSIT. One subject was excluded due to excessive motion during fMRI acquisition. Six subjects were excluded due to partially missing fMRI data. Thus, 28 CP patients (median age 34.5 [29.0–39.5] years; 17 females) were included in the study (Table 1). All subjects had undergone surgery and ten subjects had also received additional CRT (median dose 50.2 [50.0–54.0] Gy) to the area of tumor growth. At the time of investigation, the tumor location was graded retrospectively, based on each subjects’ medical records: intra-sellar growth, supra-sellar growth, supra-sellar growth toward or into the third ventricle. The latter was the criterion for hypothalamic injury [16]. Thirteen subjects were classified as having hypothalamic injury. Subjects were investigated at median 20.5 (16.3–28.8) years since first operation. At the time of investigation, 75% of the subjects received growth hormone therapy and were supplemented with growth hormone median 0.6 (0.4–0.8) mg/day. Ten females were on oral sex steroid treatment. The remaining females had normal gonadal function according to blood tests. Seven males needed testosterone replacement. Twenty-three subjects received levothyroxine. Nine subjects had normal adrenocorticotropic-cortisol axes and the reminder needed hydrocortisone. None were smokers. Comparisons were made with 29 control subjects (median age 37.0 [32.5–42.0] years; 17 females) that were matched in regard to age, gender, and smoking habits. The controls were recruited from a pool of ten potential control subjects per patient, matched for age, gender, and smoking habits, that were selected randomly from a computerized register of the population in the catchment area of the patients as previously described [17]. All subjects gave written informed consent. The study was approved by the local ethics committee (DNR 2011/769).

**Table 1:** Characteristics of the adults with childhood craniopharyngioma (CP) investigated in the present study

	All subjects (*n* = 28)	Hypothalamic injury (*n* = 13)	No hypothalamic injury (*n* = 15)
Males/Females (n)	11/17	5/8	5/10
Age at investigation (y)	34.5 (29.0–39.5)	33.0 (24.0–38.0)	36.0 (30.0–41.0)
Age at diagnosis (y)	12.0 (9.0–15.8)	13.0 (8.0–21.0)	12.0 (9.0–14.0)
Time from first operation (y)	20.5 (16.3–28.8)	18.0 (12.0–21.0)	23.0 (19.0–31.0)
CRT (n)	10	8	2
Target dose CRT (Gy)	50.2 (50.0–54.0)	50.0 (50.0–54.0)	52.1 (37.7–45.3)
Growth hormone (n)	21	10	11
Gonadal steroids (n)	19	10	9
Levothyroxine (n)	23	11	12
Cortisone (n)	19	10	9
Antidiuretic hormone (n)	23	9	14

Data are presented as median and quartile (first–third)

*CRT* cranial radiotherapy

### fMRI acquisition

MRI data were acquired on a 3T MRI scanner (MAGNETOM Skyra, Siemens Healthcare, Erlangen, Germany) equipped with a 20-channel head/neck receiver coil. A gradient-echo EPI sequence (TR/TE 1500/30 ms/ms, 25 slices, 64 dynamic scans, voxel size = 3 × 3 × 4 mm^3^) was used to acquire data during the MSIT. To perform the fMRI analyses described below, an additional T1-weighted 3D magnetization prepared gradient-echo (MP-RAGE) sequence (TR/TE 1900/2.54 ms), with 1 mm^3^ isotropic resolution, was also acquired.

### fMRI task

**Fig. 1:**
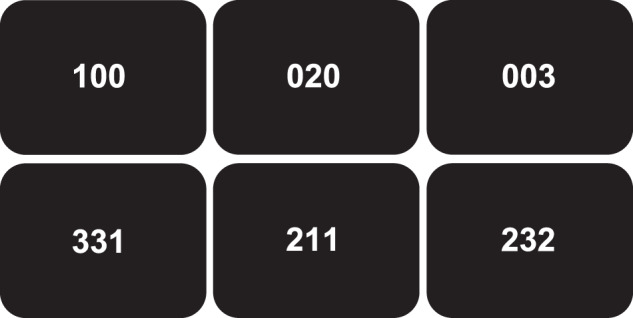
The objective during the multi-source interference task was to report, via button-press, the identity of the displayed number that differed from the other two numbers. During the control tasks (upper row), the distractors were zeros, and the target numbers (either 1, 2, or 3) were always placed congruently with their position. During the interference tasks (lower row), the distractors were either 1, 2, or 3, and the target numbers were never placed congruently with their position. The correct answer for the first column is hence ‘1’, for the second ‘2’, and for the third ‘3’

The MSIT was executed in concordance with the instructions of Bush et al. [15]. In short, the subjects were given an MRI-compatible three-button keypad and instructed that the keypad buttons represented the numbers 1, 2, and 3 from, left to right. The subjects were told to use the right index, middle, and ring finger to respond. They were also instructed that three numbers would appear in the center of the screen every few seconds. The objective was to report, via button-press, the identity of the displayed number that differed from the other two distractor numbers (Fig. 1). During the control tasks, the distractors were zeros, and the target numbers (either 1, 2, or 3) were always placed congruently with their position. During the interference tasks, the distractors were either 1, 2, or 3, and the target numbers (either 1, 2, or 3) were never placed congruently with their position. After reviewing instructions, each subject performed the MSIT once to make sure that they could perform the task correctly. Next, each subject performed the MSIT during fMRI acquisition. The subjects completed two scans each. During each scan, four 42 s blocks of the control tasks alternated with four 42 s of the interference tasks. Each block consisted of 24 three digit-number combinations. Response times (ms) and accuracy performance (%) were recorded using E-prime 2.0 (Psychology Software Tools, Pittsburgh, PA).

### Statistical analysis of behavioral performance

Data are presented as median and quartile (first–third). The interference effect is defined as the difference in response time and accuracy performance between the interference and control tasks. Comparisons were made between CP patients and controls, and the two subgroups of CP patients and controls, respectively, using the Mann–Whitney *U* test. The Wilcoxon signed-rank test was used to compare differences in response time and accuracy performance between interference and control tasks within the groups. Due to relatively small test samples, parameters could not be assumed to be normally distributed and therefore nonparametric tests were used. Calculations were made using SPSS version 26 and results were regarded as statistically significant if *p* < 0.05.

### fMRI activity analysis

Lower-level analyses generated fMRI contrasts through the subtraction of fMRI signal during the control tasks from the interference tasks for each subject, i.e., the interference effect. These contrasts were used in an additional higher-level analysis to compare group means. The CP patients were compared to controls. In addition, CP patients with and without hypothalamic injury, respectively, were compared separately to the control group. fMRI data processing was carried out using FEAT (FMRI Expert Analysis Tool) Version 6.00, part of FSL (FMRIB’s Software Library, www.fmrib.ox.ac.uk/fsl. Registration to standard space images was carried out using FLIRT and FNIRT [18–21]. The following pre-statistics processing was applied: motion correction using MCFLIRT [19]; slice-timing correction using Fourier-space time-series phase-shifting; non-brain removal using BET [22]; spatial smoothing using a Gaussian kernel of FWHM 5 mm; grand-mean intensity normalization of the entire 4D dataset by a single multiplicative factor; high-pass temporal filtering (Gaussian-weighted least-squares straight line fitting, with sigma = 45.0 s). Time-series statistical analysis was carried out using FILM with local autocorrelation correction [23]. Z (Gaussianised T/F) statistic images were thresholded non-parametrically using clusters determined by *Z* > 3.1 and a (corrected) cluster significance threshold of *p* = 0.05 [24]. Higher-level analysis was carried out using FLAME (FMRIB’s Local Analysis of Mixed Effects) stage 1 [25–27].

## Results

### Behavioral performance

Results of the analyses in terms of response time (ms) and accuracy performance (%) during the MSIT are presented in Table 2. The reaction time was significantly increased in the interference tasks as compared to the control tasks for both CP patients (861.5 [786.3–926.1] ms vs. 523.2 [485.8–576.6] ms) and controls (821.1 [770.4–891.5] ms vs. 505.9 [453.3–541.9 ms). The same was seen in the subgroup with hypothalamic injury (858.8 [789.5–919.0] ms vs. 510.6 [490.3–582.1] ms) and in those without hypothalamic injury (864.2 [780.0–948.6] ms vs. 527.8 [475.4–575.5] ms). The accuracy performance was significantly reduced in the interference tasks as compared to the control tasks for both CP patients (96.6 [93.8–98.4] vs. 100.0 [99.5–100.0]%) and controls (96.9 [94.8–97.9] vs. 100.0 [99.5–100.0]%). The same was seen in the subgroups of CP patients i.e., those with hypothalamic injury (95.3 [93.5–97.7] vs. 99.5 [99.0–100.0]%) and those without hypothalamic injury (97.9 [94.8–99.0] vs. 100.0 [100.0–100.0]%). There were no significant differences in reaction time or accuracy performance in neither the interference nor the control tasks between the CP patients, or the two subgroups, with and without hypothalamic damage, respectively, as compared to the control group. The interference effect, regarded as the difference in reaction time and accuracy performance between the control and interference tasks was not significantly different between CP patients (333.9 [287.3–367.1] ms and 3.1 [1.6–5.6]%, respectively) and controls (309.1 [276.4–361.0] ms and 2.6 [1.6–4.9]%). Similar, no significantly difference in the interference effect was seen between the subgroups; i.e., those with hypothalamic injury (332.0 [283.6–353.4] ms and 4.2 [2.3–5.7]%, respectively) and those without hypothalamic injury (355.7 [293.7–388.7] ms and 2.1 [1.0–5.2]%, respectively) compared to controls.

**Table 2:** Reaction time and accuracy performance during the interference and control tasks in the multi-source interference task (MSIT), as well as the interference effect, i.e., the difference in reaction time and accuracy performance between the interference and control tasks, for both adult childhood craniopharyngioma (CP) patients, the subgroup of CP patients with and without hypothalamic injury, respectively, and controls

	Control task		Interference task		Interference effect	
	Reaction time (ms)	Accuracy performance (%)	Reaction time (ms)	Accuracy performance (%)	Difference in reaction time (ms)	Difference in accuracy performance (%)
CP patients (*n* = 28)	523.2 (485.8–576.6)	100.0 (99.5–100.0)	861.5 (786.3–926.1)	96.6 (93.8–98.4)	333.9 (287.3–367.1)^a^	3.1 (1.6–5.6)^a^
CP patients with HI (*n* = 13)	510.6 (490.3–582.1)	99.5 (99.0–100.0)	858.8 (789.5–919.0)	95.3 (93.5–97.7)	332.0 (283.6–353.4)^a^	4.2 (2.3–5.7)^a^
CP patients without HI (*n* = 15)	527.8 (475.4–575.5)	100.0 (100.0–100.0)	864.2 (780.0–948.6)	97.9 (94.8–99.0)	355.7 (293.7–388.7)^a^	2.1 (1.0–5.2)^a^
Controls (*n* = 29)	505.9 (453.3–541.9)	100.0 (99.5–100.0)	821.1 (770.4–891.5)	96.9 (94.8–97.9)	309.1 (276.4–361.0)^a^	2.6 (1.6–4.9)^a^

Data are presented as median and quartile (first–third)

*CP* craniopharyngioma, *HI* hypothalamic injury

^a^Significant difference (*p* < 0.05) between interference and control tasks

### fMRI activity

**Fig. 2:**
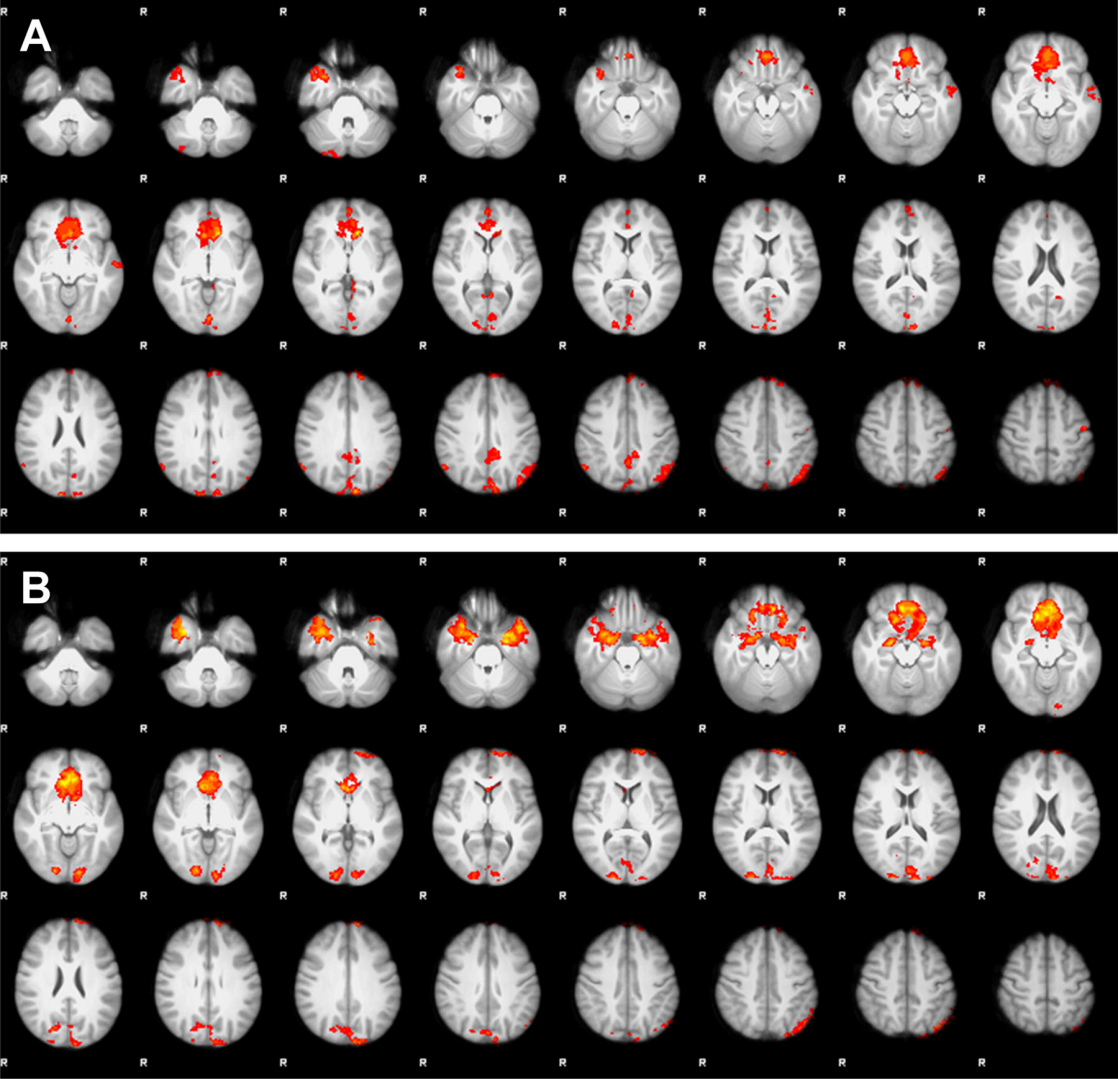
Mean difference in fMRI activity between the interference and control tasks in the multi-source interference task, i.e., the difference in neuronal activity required to perform the additionally more cognitively demanding interference tasks compared to the control tasks, revealed fMRI activity pattern in the cingulo-fronto-parietal attention network as excepted but showed no significant differences between the adult childhood craniopharyngioma (CP) patients (**A**) and controls (**B**), the CP patients with and without hypothalamic injury (not shown here), respectively, and controls

Results of the analyses of fMRI activity during the MSIT are shown in Fig. 2. The difference in fMRI activity required to perform the more cognitively demanding interference tasks compared to the control tasks, demonstrated fMRI activity in the CFP attention network, in both the CP patients, the two subgroups of CP patients with and without hypothalamic injury, respectively, and in the control group. Comparisons between CP patients and controls, CP patients with hypothalamic injury and controls, and CP patients without hypothalamic injury and controls showed no significant differences in fMRI activity between any of the groups.

## Discussion

The aim of this study was to assess cognitive interference processing in adults with childhood CP with the MSIT during fMRI acquisition in terms of behavioral performance and fMRI activity. Our results show that there was a difference in both reaction time and accuracy performance between the interference and control tasks for both the CP patients and controls, which confirms the validity of the method. However, there were no differences in reaction time, accuracy performance, or fMRI activity between any of the groups in performing the interference tasks.

A significant difference in reaction time as well as in accuracy performance was recorded between the more cognitively demanding interference tasks and the control tasks, for both the CP patients, the subgroups of CP patients with and without hypothalamic injury, respectively, and the controls. This indicate that the MSIT was performed according to instructions described in the literature [14, 15]. Nevertheless, there were no differences in neither reaction time nor accuracy performance between any of the investigated groups. The interference effect, i.e., the difference in reaction time and accuracy performance between the interference and control tasks, also did not differ between any of the investigated groups.

The interference effect was additionally studied regarding fMRI activity and revealed activation in the CFP attention network as expected in both groups, suggesting that the results are reliable. Notably, the difference in fMRI activity that was needed to solve more cognitively demanding interference tasks compared to the control tasks did not differ between any of the investigated groups. This suggests that the CP patients performed cognitive interference processing on a comparable level to controls, without any compensatory activation in the CFP attention network.

The MSIT has previously been used to study alterations in behavioral performance and fMRI activation in different conditions, commonly within the field of psychiatry [28–32], but also in patients with heart disease [33]. The results are highly varying and only some studies acquired fMRI data during the MSIT whereas most studies have only presented results on behavioral performance. In previous studies using fMRI, decreased fMRI activity in the rostral anterior cingulate/medial prefrontal cortex and the precuneus/posterior cingulate cortex have been seen in patients with schizophrenia and increased fMRI activity in the medial frontal cortex in patients with obsessive-compulsive disorder but no differences in behavioral performance compared to controls [28, 29]. These somewhat ambiguous results could be due to different conditions affecting different parts of the brain and that structures within the investigated area are responsible for different functions. In other words, increased activity in one area could result in the same altered behavioral performance as decreased activity in another area.

Neuroimaging studies on childhood CP are scarce and comparisons of the results are difficult to interpret, largely due to methodological variations and limitations such as small samples, different treatment protocols, and different follow-up times (Table 3). A few previous studies have demonstrated that white matter integrity in the investigated areas correlated negatively to given radiation dose, reduced gray and white matter volumes in the limbic areas, and have shown a negative correlation between long-term memory and gray matter in the posterior cingulate cortex [10, 34]. In another previous study, using a subset of patients included in the present study, microstructural white matter integrity was assessed using DTI and tractography and an association between decreased integrity in the dorsal cingulum and a decline in episodic visual memory, visuospatial abilities, executive function, attention, and processing speed was found [11]. Furthermore, the same study found an association between decreased integrity in the ventral cingulum and a decline in episodic visual memory, and an association between decreased integrity in the uncinate fasciculus and a decline in semantic memory in adult childhood CP patients [11].

**Table 3:** Previous neuroimaging studies on craniopharyngioma patients

Study	Subjects (*n*)	Age at investigation (y)	Follow-up time (y)	CNS treatment	Neuroimaging and field strength	Findings
Roth et al., 2012 [12]	4	13–17 years	>1	Surgery CRT	fMRI to evaluate activity in correlation to visual food cues at 3 T	Increased activity in medial OFC following meal
Özyurt et al., 2014 [13]	10	Median 17.8	>4	Surgery CRT	fMRI test for emotional face recognition at 1.5 T	Altered activity in PFC during memory retrieval
Uh et al., 2015 [34]	51	Median 9.2 (range 2.1–19.3)	3	Surgery CRT (proton radiation equivalent to 54 Gy)	Atlas-based ROI analysis of DTI at 1.5 T	Negative association between WM integrity and radiotherapy
Özyurt et al., 2017 [10]	11	17.4 (IQR 8.6–26.2)	~ 5–10	Surgery	Voxel-based morphometry at 1.5 T	Reduction of GM and WM volumes in limbic areas
Fjalldal et al., 2018 [11]	41	≥17	Median 35 (range 17–56)	Surgery CRT (50 [range 35–55] Gy)	DTI and tractography at 3 T	Negative association between WM integrity in the cingulum and cognitive functions

*CNS* central nervous system, *CRT* cranial radiotherapy, *fMRI* functional magnetic resonance imaging, *GM* gray matter, *OFC* orbitofrontal cortex, *PFC* prefrontal cortex, *WM* white matter

Only two previous studies have used fMRI to study cognitive impairment in childhood CP patients. These studies demonstrated lower fMRI activity during the premeal test and higher fMRI activity during the post-meal test as compared to controls [12], and differential recruitment of fronto-limbic brain regions during emotional face recognition [13]. Even though the hypothalamus is partially connected to the limbic system as well as partially to the CFP attention network, investigated in the present study, the investigated cognitive domains, chosen for evaluation, differs between the studies and hence comparisons of the results to the present study are not easily interpreted [35, 36].

In comparison to previous studies on CP survivors that have demonstrated cognitive deficits, and functional and structural brain alterations, albeit using slightly different techniques and testing slightly different cognitive domains and neuroanatomical structures [7–13], the results of the present study may appear somewhat contradictory. However, not all previously investigated cognitive domains and/or neuroanatomical structures were affected. For example, one study using a sub-sample of the same CP patient group as in the present study found cognitive deficits in semantic, episodic, and visual memory, but no deficits in working memory, executive function, attention or processing speed [11]. In this context, the results of the present study may not be that surprising since cognitive interference processing is more related to executive function and attention than to semantic, episodic, and visual memory because it tests the ability to select and organize relevant information and to suppress irrelevant information. The present results are also interesting because the CFP attention network, situated in an area where microstructural alterations in major white matter tracts have previously been reported [11], have never been investigated in CP survivors before. Thus, the results of the present study should therefore rather be considered as another piece of information required to better characterize this condition.

To our knowledge, this is the first study that has used the MSIT in combination with fMRI to study cognitive interference processing in adults with childhood CP. Reaction times, accuracy performance, and fMRI activity indicate that the MSIT was performed correctly and that the results are reliable. Still, there are some limitations to this study. Firstly, the study groups were, due to the rareness of the disease and the long follow-up time, relatively small and may have underpowered the study, leading to discarded true differences between the investigated groups. Secondly, a possible selection bias might have occurred when subjects that were unable to perform the task were excluded.

In conclusion, adults with childhood CP performed cognitive interference processing equally well as controls in terms of response times and accuracy performance and did not exhibit altered fMRI activity in the CFP attention network during the process. This was also true for the two subgroups with and without hypothalamic injury. However, this does not exclude deficits in other cognitive domains or alterations in other functional networks. Further studies are needed to map what cognitive domains and functional networks are affected/unaffected in CP survivors in order to better characterize the condition and to optimize treatment and support for these individuals.

## Data Availability

Supplementary material is available upon request from the corresponding author.

## Code availability

Scripts containing information of settings used for data analysis is available upon request from the corresponding author.
